# Chemiluminescence Detection in the Study of Free-Radical Reactions. Part 1

**DOI:** 10.32607/actanaturae.10912

**Published:** 2021

**Authors:** L. A. Romodin

**Affiliations:** Moscow State Academy of Veterinary Medicine and Biotechnology – MVA named after K.I. Skryabin, Departmental affiliation is Ministry of Agriculture of the Russian Federation, Moscow, 109472 Russia

**Keywords:** free radical reactions, apoptosis, ferroptosis, chemiluminescence, lipid peroxidation, reactive oxygen species

## Abstract

The present review, consisting of two parts, considers the application of the chemiluminescence detection method in evaluating free radical reactions in biological model systems. The first part presents a classification of experimental biological model systems. Evidence favoring the use of chemiluminescence detection in the study of free radical reactions, along with similar methods of registering electromagnetic radiation as electron paramagnetic resonance, spectrophotometry, detection of infrared radiation (IR spectrometry), and chemical methods for assessing the end products of free radical reactions, is shown. Chemiluminescence accompanying free radical reactions involving lipids has been the extensively studied reaction. These reactions are one of the key causes of cell death by either apoptosis (activation of the cytochrome *c *complex with cardiolipin) or ferroptosis (induced by free ferrous ions). The concept of chemiluminescence quantum yield is also discussed in this article. The second part, which is to be published in the next issue, analyzes the application of chemiluminescence detection using luminescent additives that are called activators, a.k.a. chemiluminescence enhancers, and enhance the emission through the triplet–singlet transfer of electron excitation energy from radical reaction products, followed by light emission with a high quantum yield.

## INTRODUCTION

Biochemiluminescence is the generation of photons in biological systems. There is also the term “bioluminescence,” which is, strictly speaking, meaningless, since it stands for light emission produced by chemical reactions in living organisms. The luminescence in these systems results from reactions involving free radicals. Chemiluminescence detection is used to study the reactions and the impact of various factors such as antioxidants on this process. Prior to directly describing chemiluminescence and its mechanisms of occurrence in biological systems, several words should be said about the systematization of biological model systems.

## BIOLOGICAL MODEL SYSTEMS IN THE STUDY OF FREE RADICAL REACTIONS

An experimental model system is a material system that, once affected by a physical, chemical, biological or any other factor, can provide information about the effect of the factor on the original system. Here, we present a classification of the experimental model systems used in biological studies.

A. Biological model systems:

A1. Laboratory animals. This model most fully represents the properties of the human body. However, the taxonomic characteristics of the animals used (e.g., the ability to synthesize vitamin C) should be taken into account. This will allow for understanding how the result obtained in this model can be applied to the human body. An example is the study of free radical processes in mice carried out by the M.V. Listov research team [1, 2] and a model of acetaminophen- (paracetamol-) induced liver cirrhosis in rats [3];

A2. Animal embryos. The main difference of this model from the previous one is that it allows for reducing the experimental time and studying a more complete set of effects thanks to the fact that regulations regarding laboratory animals do not apply to embryos at early developmental stages. An example is the work on the effects of vitamin E deficiency and hypervitaminosis on *Brachydanio rerio *(zebrafish) parents studied in fish embryos [4];

A3. Neuromuscular agent. The free radical nature of excitation and inhibition in neuronal tissue was demonstrated using this model [5];

A4. Cell cultures. This model is used to determine the formaldehyde level by registering chemiluminescence enhanced by coumarin derivatives under conditions of artificially induced stress [6];

A5. Mitochondrial culture. This model allows for the study of mitochondrial processes. An example is the works on chemiluminescence detection in mitochondrial suspension conducted by Yu.A. Vladimirov* et al. *[7, 8, 9]. The results of those studies suggest that peroxidation of lipids in mitochondrial membranes is initiated in condition of deficiency of the enzymes that catalyze β-oxidation of fatty acids. Another example is an isolated culture of plant plastids: e.g., chloroplasts [10];

A6. Tissue samples. In the study of tissues obtained directly from animals, a laboratory animal serves as an experimental model. Biochemiluminescence was first detected in a tissue sample [11]. The method of detecting the chemiluminescence of blood and its fractions is used in many studies [12, 13, 14, 15, 16];

A7. Fungi model. The most commonly used experimental model is baker’s yeast (*Saccharomyces cerevisiae*). This model was used to study oxidative stress by detecting chemiluminescence [17];

A8. Plant models. This group of models includes both whole plants, seedlings, individual organs, and cultures of plant cells and tissues. An increase in the concentration of the superoxide anion radical upon enhanced activity of lipoxygenases was shown in bean cotyledons [18]. Another example is the use of the chemiluminescence detection method in the study of a peptide ligand binding to a cell receptor [19].

A large group of models called molecular models can be also distinguished; it includes two groups of systems.

B. Conditionally biological experimental models:

B1. Models based on biological molecules isolated from living organisms. Examples include cytochrome *c* and cardiolipin isolated from animals [20] and *Escherichia coli *DNA [1];

B2. Molecular models based on biological molecules isolated from living organisms and artificially synthesized molecules identical to them. Examples are the study of the participation of coumarin derivatives in the reaction catalyzed by the cytochrome *c *complex with cardiolipin using cytochrome *c *isolated from the horse’s heart and artificially synthesized tetraoleyl cardiolipin [21];

C. Models based on synthetic polymers and low-molecular- weight organic compounds. Technically, these models cannot be considered biological. However, some data obtained with their use can be applied to living systems. In addition, these models are often the most suitable choice for studying the basic principles of free radical reactions:

C1. A molecular model that uses biomolecules and their non-biological analogue. For instance, the dodecyl sulfate anion is used as a cardiolipin analogue to study changes in cytochrome *c *properties upon its binding to phospholipids [22]. This model makes it possible to study the complex of cytochrome *c *with cardiolipin, which induces peroxidation of lipids in mitochondrial membranes, resulting in the activation of apoptosis through the mitochondrial pathway [23];

C2. Molecular model using a synthetic polymer. This model was used to study chemiluminescence produced by polymer decomposition [24] and the kinetics of alkyl radical decay in polyethylene [25];

C3. Molecular model based only on low-molecular- weight organic compounds. The use of this model made it possible to obtain data on the nature of the chemiluminescence caused by reactions involving hydrocarbon radicals through the action of the products of thermal decomposition of α_1_,α_2_-azobisisobutyronitrile [26]. Hydrocarbons can be considered a very convenient model for studying free radical reactions involving lipids, since the tails of lipid molecules are hydrocarbons. The results of such work have been published [26, 27] and contributed to the study of the mechanisms of lipid peroxidation [28, 29, 30, 31].

## CHEMILUMINESCENCE AND ITS MECHANISM

Emission of light of very low intensity by biological objects was first noticed at the end of the first third of the previous century: V.V. Lepeshkin discovered the emission from photographic plates lying on biological samples. He considered this radiation to be ultraviolet emitted during protoplast coagulation upon cell death and called it necrobiotic radiation [32, 33]. A.G. Gurvich, who detected luminescence of a suspension of fission yeast, suggested the signaling role of the luminescence of biological samples in the ultraviolet spectral region. He further called this luminescence “mitogenetic radiation” [34].

Subsequenly, with the help of photomultipliers, in the third quarter of the 20th century visible light emission of extremely low intensity produced by biological objects of plant origin [36] and animal tissues [11] was detected and called *ultraweak chemiluminescence* in the English language literature [35]. Chemiluminescence of intact tissues, mitochondria [7, 8, 9], and chloroplasts [10] was discovered. In the early 1970s, R. Allen discovered chemiluminescence of human blood leukocytes during bacterial phagocytosis [37, 38]. This discovery made it possible to use chemiluminescence as a clinical method for determining immunoreactivity.

Chemiluminescence is luminescence caused by the transition of various metabolites of free radical reactions from an electronically excited state (EES) to the ground state [39, 40].

**Free radical reactions in biological systems**

A free radical is a particle with a free valence that is due to the presence of an unpaired electron. M. Gomberg was the first to describe radicals at the beginning of the 20th century [41, 42, 43]. Free radicals are highly reactive, meaning that they are chemically unstable and have a short lifetime. The molecular structure of a radical can affect its stability. For example, methyl groups [44, 45] and an iminoacetyl group in the *para* position [44] stabilize the quinone radical.

Radical forms of the respiratory chain components were discovered in the middle of the 20th century: single-electron energy transfer was described [46, 47, 48]. Previously, redox reactions in biological systems were believed to involve only the release and acceptance of two electrons simultaneously [31].

One of the most important radicals in oxidative stress is the superoxide anion radical (O_2_^●−^), resulting from the interaction between a semiquinone radical (semi-reduced ubiquinone) and molecular oxygen at the inner side of the mitochondrial membrane, in the respiratory complexes III [49] and I [29], and in the cytoplasm (in the NADPH oxidase complex in the endoplasmic reticulum membrane or plasmalemma) [50, 51]. In addition, the superoxide radical is formed during the oxidation of hemoglobin to hemin [2]. The resulting superoxide radical participates in neurohumoral regulation [1, 2, 5, 52]. M.V. Listov *et al. *found that the superoxide anion radical formed in the blood promotes the generation of cell surface potentials, acting as a trigger for effectors [5]. In particular, the superoxide radical contributes to automatic contractions of the myocardium, acting on the sinoatrial node of the cardiac conduction system [52] and serving as a major factor in the depolarization and hyperpolarization of the cell membrane. Thus, the superoxide radical triggers the mechanisms of excitation and inhibition on the surface of conducting fibers [5]. Along with nitrogen monoxide formed by NO-synthases, the superoxide anion radical was called primary in the classification proposed by Yu.A. Vladimirov [29]. This term indicates that formation of both radicals is catalyzed by enzymatic systems [29, 53].

**Fig. 1:**
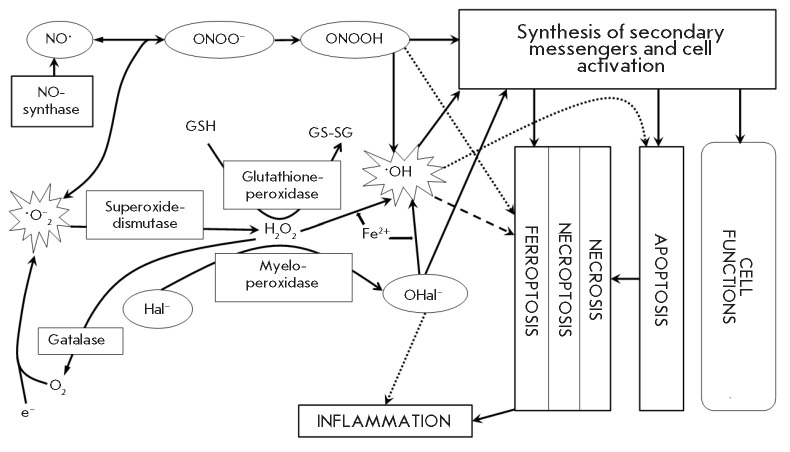
Metabolic pathways involving free radicals [29, 54, 55, 56, 57]

Primary radicals form the following molecular products: O_2_^●−^ is either converted to hydrogen peroxide by superoxide dismutase or reacts with NO^●^ producing the toxic peroxynitrite ion ONOO^•P^ [54]. Superoxide can also reduce the ferric iron in ferritin and the iron-sulfur clusters of electron transport chains to a bivalent ion, which further reacts with hydrogen peroxide or hypochlorite to form an extremely reactive hydroxyl radical (^●^OH) and can branch lipid oxidation chains by reacting with lipid hydroperoxides. The hydroxyl radical can activate lipid peroxidation with formation of lipid radicals [29]. The resulting reactive oxygen and nitrogen species, as well as hypochlorite at low concentrations, act as secondary messengers. When cellular antioxidant systems are impaired (the major role is played by glutathione and glutathione peroxidase [56]), these radicals induce oxidative stress, leading to wither apoptosis [23, 58] or ferroptosis [59, 60, 61] through lipid peroxidation. It should be noted that lipid peroxidation leading to apoptosis is usually induced by cytochrome *c* complexed with cardiolipin. Binding of cytochrome *c *to cardiolipin changes its conformation so that the protein acquires the ability to catalyze lipid peroxidation [62, 63, 64]. Ferroptosis is induced by initiation of the Fenton reaction by Fe^2+^ ions, followed by lipid peroxidation initiated by hydroxyl radicals [59, 60, 61]. Both hydroxyl and lipid radicals are secondary in the Yu.A. Vladimirov classification [53]. The diagram in *Fig. 1* shows major metabolic pathways involving free radicals. It should be noted that there is no unified system of terms describing free radical reactions in biological systems and oxidative stress.

**Detection of free radicals in biological systems, intrinsic chemiluminescence**

The method of chemiluminescence detection makes it possible to estimate the rate of free radical formation [28, 31]. This physical method is used to study free radical reactions together with chemical methods for detecting the molecular products of radical reactions. The most common marker of free radical reactions and the state of oxidative stress is one of the products of lipid peroxidation, malondialdehyde (MDA), whose concentration is determined using thiobarbituric acid (TBA) [65, 66]. In order to obtain more reliable results, the concentration of Schiff bases [67, 68], diene [69, 70], and triene [67] conjugates should be also measured. Other methods are based on the use of radical scavengers: antioxidant enzymes such as catalase (H_2_O_2_) [71] and superoxide dismutase (O_2_^●−^) [69], phenolic antioxidants for hydroxyl/lipid radicals, and other organic molecules [71]. The main disadvantage of chemical methods is the impossibility of determining the nature and concentration of free radicals [29].

The method of electron paramagnetic resonance (EPR), developed in the middle of the 20th century [72], makes it possible to detect and identify many radicals by analyzing the hyperfine structure of EPR signals [73, 74]. However, the use of EPR is hampered by the short lifespan and, thus, low concentration of free radicals [75]. For this reason, only the use of a flow-through system with a high consumption of reagents made it possible to detect radicals formed in the reaction between Fe^2+^ cations and lipid hydroperoxides [76]. Reagent consumption can be reduced by using spin traps [1, 77], which, however, can affect the biochemical reactions in the system, and also be destroyed in some of them [29]. Free radical reactions in heme-dependent exophthalmos were studied using EPR and infrared (IR) spectroscopy [1]. Another physical method, spectrophotometry, should be also mentioned. This method was used to determine the concentration of oxidation products when studying the mechanisms of heteroauxin (β-indoleacetic acid) oxidation by horseradish peroxidase and tobacco anionic peroxidase [78]. The concentration of lipid peroxidation markers in the overwhelming majority of cases is also determined using spectrophotometry. Coumarin derivatives used as a luminescent additive to assess the peroxidase properties of the cytochrome *c *complex with cardiolipin were studied using spectrophotometry and chemiluminescence detection [21].

The method of chemiluminescence detection makes it possible to study the intensity of reactions involving short-lived radicals. This is possible thanks to the large amount of energy produced in a radical reaction and partially released in the form of photons [40].

Here we present widely available information on the kinetics of reactions accompanied by chemiluminescence. In these reactions, the initial substances R form free radicals R^●^, which can generate electronically excited products P* in a subsequent reaction, which, in turn, when converted to the ground state P, can emit a photon (*hν*). The chance of formation of an EES product is very high if the activated complex of reagents and reaction products has states with different multiplicities [40]. For the convenience of further description of the processes under consideration, we present the general scheme of a chain reaction with the formation and participation of free radicals, followed by photon emission:

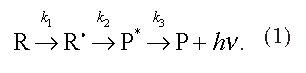



It should be noted that, in most cases, the chemiluminescence spectrum does not correspond to the fluorescence spectrum of the product P^*^ but corresponds to its phosphorescence spectrum [79]. This clearly indicates that products P^*^ are in a triplet excited state.

The intensity of chemiluminescence (*J*) is proportional to the rate of the third reaction in the abovementioned scheme (1): *J*∝*k*_3_[P*].

Due to the high rate of free radical conversion to reaction products, the steady state, when the rates of all reactions in the reaction chain are equal, is quickly established in the system. Thus, the luminescence intensity is proportional to the rate of free radical formation* v*1 (reaction with the rate constant *k*1). Hence, the chemiluminescence intensity is also proportional to the steady-state concentration of free radicals, which can be determined based on the rate of their formation and the rate constant of conversion to EES products [40, 80]:

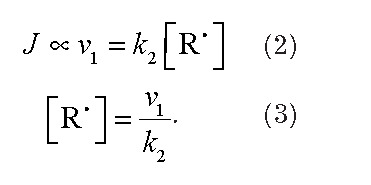



It is important to note that both the EPR method and fluorimetry/spectrometry are used to determine the concentrations of substances, which are free radicals [R^•^] in our case. The [R^•^] value, and thus the recorded signal, decreases with the growth of radical reactivity; i.e., with an increase in *k*_2_. Therefore, active radicals, even with an extremely high production rate, are not detected by EPR because of the high *k*_2_ value: i.e. high rate of their conversion to reaction products. However, the chemiluminescence intensity does not depend on the concentration of radicals but rather on the rate of free radical reactions. For this reason, this method can be used to detect even the most reactive radicals at extremely low concentrations [80].

**Quantum yield of intrinsic chemiluminescence**

Two concepts of the quantum yield should be mentioned when considering chemiluminescence: the quantum yield of excitation (*Q*_ex_), which is the ratio of reaction product molecules in EES to the total number of reaction product molecules; and the luminescence quantum yield (*Q*_lum_), which is the ratio of molecules in EES emitting a photon to the total number of molecules in EES. The total yield of luminescence, namely chemiluminescence (*Q*_ChLum_), is equal to their multiplication:* Q*_ChLum_ = *Q*_ex_ ∙ *Q*_lum_ [40].

Let us consider the reactions presented in scheme (1) with the rate constants *k*2 and *k*3 in more detail:

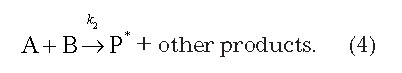



A chemiluminescent reaction [40].

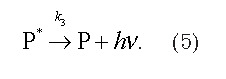



A luminescenct reaction [40].

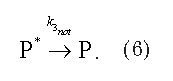



The quantum yield *Q*_lum_ of reaction (5) is the quantum yield of the product photoluminescence, which is close to zero in most biochemical reactions. However, the quantum yield *Q*_ex_ in the case of formation of EES products is also extremely low, since most chemical reactions in aqueous solutions at ambient temperature result in the formation of unexcited molecules in the ground electronic state [29] (“other products” in reaction (4) with the constant *k*2). The total quantum yield of chemiluminescence evaluating the rate of free radical formation is calculated using the following formula:* Q*_ChLum_ = *Q*_ex_ ∙*Q*_lum_ [40]. This luminescence is called superweak due to such a low value of the quantum yield of biochemiluminescence [31, 81].

The quantum yield value, and hence, the resulting chemiluminescence intensity, can be calculated using the formulas [40]:

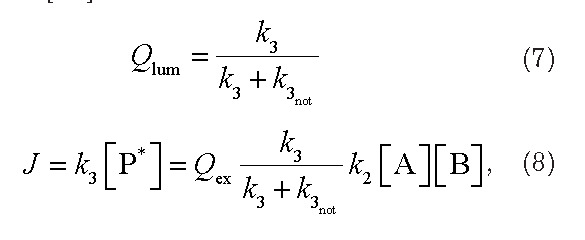



where *k*_3_ is the rate constant of reaction (5), *k*_3not_ is the rate constant of reaction (6), *k*_2_ is the rate constant of reaction (4), and *J *is the chemiluminescence intensity.

Apparently, not every light quantum entering the luminometer is capable of ejecting an electron from the photocathode of the photomultiplier tube [31]. Therefore, the software of modern luminometers takes into account the light collection coefficient (the ratio of quanta reaching the photocathode to the total number of quanta emitted by the system [82]) and the quantum yield of the photocathode (the ratio of electrons ejected from the cathode to the number of quanta reaching the cathode).

**Chemiluminescence mechanism in the peroxidation of biological molecules**

Lipid peroxidation is one of the main processes contributing to ferroptosis [60, 61, 83] and apoptosis through the mitochondrial pathway [23]. Therefore, most attention in the study of these processes is focused on radical reactions involving lipids. However, the scheme describing lipid radical reactions accompanied by chemiluminescence is generally valid and can be applied to chemiluminescent reactions involving proteins, as shown by I.I. Sapezhinskij and E.A. Lissi [75, 84, 85, 86], and nucleic acids in solutions exposed to low-frequency electromagnetic radiation [87, 88]. It should be noted that, for luminescence to occur, the energy yield of the reaction must be ≥ 40 kcal/mol (167.5 kJ/mol) [40]. The mechanisms of luminescence were initially discovered and studied in model systems based on synthetic polymers [24, 89] and low-molecular-weight organic compounds [26, 90, 91]. For instance, alkyl radical decay in polyethylene was studied [25] and the results of a spectrometric study of the chemiluminescence accompanying the oxidation of polycarbonate, polystyrene, and polyethyl methacrylate by the products of thermal decomposition of dicyclohexylperoxydicarbonate with the total quantum yield of chemiluminescence equal to 10-9 were published [24].

Lipid peroxidation, which mostly involves polyunsaturated acyl chains, is presented not as a single reaction, but a cascade of branched chain reactions [92, 93, 94]. Below is the detailed scheme of reaction (4) with the overall rate constant *k2*:

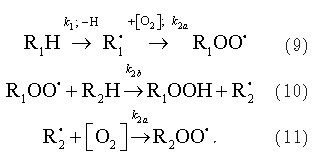



Lipid hydroperoxides ROOH very easily become the source of new lipid oxidation chains, according to the general principles of such reactions [95, 96]:

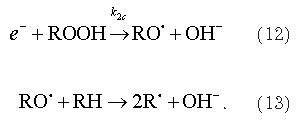



Formation of oxygen radicals is a key step in a cascade of reactions producing chemiluminescence. Despite the well-known fact that molecular oxygen is a luminescence quencher [97], the presence of oxygen in a system with proteins and hydrocarbons enhances the chemiluminescence intensity, as shown in the middle of the 20th century [26, 40, 75, 80, 90, 98]. This allows one to assume that the excited particles that ultimately emit light result from the recombination of oxygen radicals. It should be also noted that, in addition to proteins and hydrocarbon groups, luminol can also serve as a substrate for oxidation followed by photon emission [99, 100]. However, the resulting luminescent product is in a singlet but not triplet EES, which is typical of excited products of free radical reactions involving hydrocarbon groups. Luminol is widely used as an additive enhancing the chemiluminescence intensity.

The chemiluminescence accompanying lipid peroxidation reactions is caused by the disproportionation of ROO• radicals [27, 90]. Generally speaking, this process can be described as follows [90]:

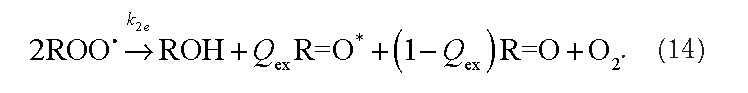



The mechanism of disproportionation of peroxyl radicals with the formation of a carbonyl compound, alcohol, and an oxygen molecule was first described by G.A. Russell [101] and later named after him. The reaction (14) is termination of the radical oxidation chain, while reaction (10) is a chain extension reaction. G.A. Russell determined the average ratio of the rate of reaction (10) to the rate of reaction (14), which is equal to 7.4 for the hydrocarbon model system [101].

Reaction (14) is a second-order reaction. Thus, it is described by a known mathematical equation:

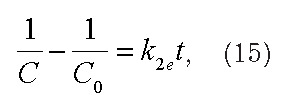



where *t *is the time from the beginning of the reaction,* C *and *C*0 are concentrations of ROO• radicals at time* t *and at the beginning of the reaction, respectively. However, M. Dole [102] states that some ROO• radicals in the system may not undergo disproportionation. The concentration of these radicals is further denoted by letter A. According to [102], the resulting formula for (15) is the following (the equation is presented in two forms for convenience):

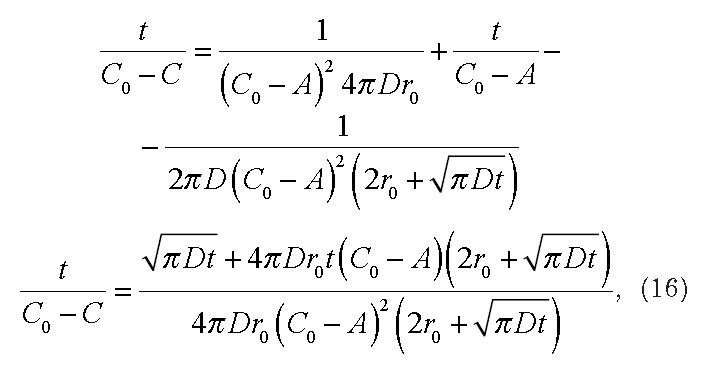



where *r*0 is the distance between radicals they react within, and *D *is the sum of diffusion coefficients of the reagents.

Photon emission occurs during the transition of ketone formed in reaction (14) from triplet EES to the ground state:

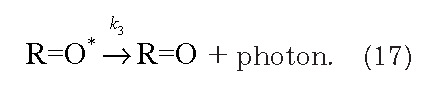



The emitted light has a maximum intensity in the region of 450–550 nm [103].

**Fig. 2:**
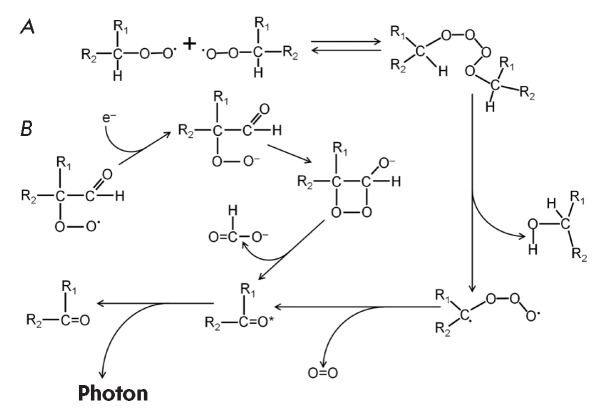
The main mechanisms of photon emission in lipid oxidation [81, 102, 104, 107]. (*A*) –disproportionation of peroxyl radicals. (*B*) – formation and decomposition of the dioxetane group (dioxetanone is presented in the diagram)

Reaction (14) proceeds with tetroxide formation, followed by its decomposition to alcohol and a diradical due to mechanical stress in the molecule skeleton: this is the time point when electrons are separated in the molecule. Next, an oxygen molecule is released and a triplet EES ketone is generated [27, 80]. However, there is a high chance that tetroxide can decompose again to two lipid peroxyl radicals. This is supported by the fact that the diffusion rate constant for these radicals is orders of magnitude higher than the rate constant of their disproportionation [27]. A graphic representation of the Russell mechanism is presented in *Fig. 2A*. The resulting oxygen can be in singlet EES. According to [104], the quantum yield of O_2_ excitation is ≈11%. Luminescence with a maximum at 634 and 703 nm is observed upon transition of oxygen to the ground state [103, 105].

Due to extremely low values of the quantum yields of formation of excited ketone molecules and their luminescence (in this case, phosphorescence), the total quantum yield of chemiluminescence is only 10–8 [80].

The relationship between the concentration of lipid peroxyl radicals and luminescence intensity *J *is determined by the equation [24, 40]:

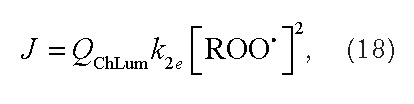



where *J *is the total light input at all wavelengths and in all directions and *Q*_ChLum_ is the quantum yield of chemiluminescence.

Apart from the Russell mechanism, there is another path of formation of carbonyl compounds in triplet EES: decomposition of the dioxetane group resulting from peroxide cyclization [86]. This process is presented graphically in *Fig. 2B*.

E.J. Bechara *et al. *investigated the mechanisms of dioxetane formation and decomposition [103]. The obtained data showed that, in addition to the classical non-radical decomposition of dioxetane to two carbonyl compounds in a triplet EES and the ground state, a radical containing a carbonyl group is formed instead of the second compound, as well as either lipid peroxide or lipid peroxyl radicals. Having avoided the Russell mechanism, these radicals can form a lipoxyl radical RO^•^, which can convert to an alkyl radical and a carbonyl compound or a radical with a either oxetane or oxirane structure, which rapidly decomposes, producing a tertiary radical bound to an alkoxy group [103]. The review by G. Cilento and W. Adam [106] presents various mechanisms of production of dioxetanes, with their subsequent cleavage to an excited product. In addition to the classical reaction scheme, the mechanism of aldehyde oxidation by oxygen through the formation of dioxetane, followed by the production of formic acid and excited aldehyde in the form of the next lower homolog, was shown [106]. The mechanism of formation of an excited ketone during oxidation and decomposition of diethylstilbestrol and other similar mechanisms were also described. Dioxetane can result from the oxidation of a phenol radical, which is produced during the interaction between phenol and a lipid peroxyl radical, by oxygen. This reaction is part of the mechanism of action of phenolic antioxidants [107]. Other ways of formation of excited products, such as recombination of two tertiary alcohol α-radicals, formation of excited products upon “sticking” of radicals due to free valences, formation of an excited ketone upon dehydration of hydrocarbon hydroperoxide (including lipid hydroperoxides), etc., were also presented [107].

Let us return to lipid peroxidation. The rate of peroxide oxidation is the rate of formation of the products of lipid oxidation by hydroperoxide in reaction (10) with the rate constant *k*_2b_:

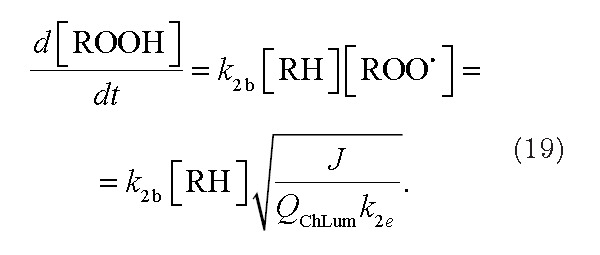



Hence, the peroxidation rate is to a certain extent proportional to the steady-state concentration of free radicals in the system and depends on the chemiluminescence intensity. Therefore, measuring the chemiluminescence intensity allows one to assess the changes in the lipid peroxidation rate over time and, thus, study the kinetics and the mechanism of this process [24].

The described relationship between the intensity of the intrinsic chemiluminescence accompanying free radical oxidation of lipids and the rate of this oxidation was confirmed by the study of successive stages of chemiluminescence in model systems containing lipids (liposomes and mitochondria) with the addition of salts dissociating to Fe^2+^ cations [108, 109]. A study of the kinetics of such chemiluminescence with determination of the level of oxygen consumption, Fe^2+^ to Fe^3+^ oxidation, and mathematical modeling of reactions [110] made it possible to determine the equations of the lipid oxidation cascade, identify the rate constants of its main reactions, and also study the effect of various antioxidants on it. The method of chemiluminescence detection is a convenient tool to study lipid peroxidation. This method was widely used by R.F. Vasil’ev [111, 112, 113, 114, 115, 116], Yu.A. Vladimirov [62, 117, 118, 119], A.I. Zhuravlev [31], and other researchers [17, 30, 120, 121, 122, 123, 124, 125, 126, 127].

As things stand, the study of the kinetics of lipid peroxidation caused by free iron ions is becoming relevant again. This is due to the discovery of another type of programmed cell death in 2012: ferroptosis [61], which is necrosis-like cell death caused by the oxidation of mitochondrial structures, primarily membranes, induced by iron ions through the Fenton reaction [59, 83, 93].

Detection of intrinsic chemiluminescence is used in the study of various biological model systems [29, 128, 129]. In addition to lipid peroxidation, NO synthesis also causes tissue chemiluminescence, as shown by J.F. Turrens *et al. *in perfused lung and model systems [130, 131]. Interaction of peroxynitrite with proteins is another source of chemiluminescence [132], with interaction of peroxynitrite with tryptophan making the greatest contribution to luminescence, while reaction with phenylalanine provides a somewhat smaller yield [131]. This method for detecting intrinsic chemiluminescence has been successfully used in the study of the peroxidation of lipids comprising low-density lipoproteins in blood plasma stimulated by neutrophils [133].

However, the intensity of intrinsic chemiluminescence is extremely low in the majority of cases [29, 31, 134], which significantly complicates its detection. In addition, a study often requires the analysis of specific radicals. For example, lipid peroxidation reactions require an assessment of the presence of lipid radicals in the system. However, the method of chemiluminescence detection has no specificity [29]. Therefore, most studies require the use of specific luminescent additives that enhance the signal through a migration of the electronic excitation energy from the molecules resulting from free radical reactions to them, followed by the emission of photons with a higher quantum yield than that of the products. These substances can be called enhancers or chemiluminescence activators; they will be discussed in the next part of the review.

## Abbreviations

MDA: malonic dialdehyde
EES: electronically excited state
EPR: electron paramagnetic resonance
